# RNA-Binding Protein Musashi1 Modulates Glioma Cell Growth through the Post-Transcriptional Regulation of Notch and PI_3_ Kinase/Akt Signaling Pathways

**DOI:** 10.1371/journal.pone.0033431

**Published:** 2012-03-12

**Authors:** Jun Muto, Takao Imai, Daisuke Ogawa, Yoshinori Nishimoto, Yohei Okada, Yo Mabuchi, Takeshi Kawase, Akio Iwanami, Paul S. Mischel, Hideyuki Saya, Kazunari Yoshida, Yumi Matsuzaki, Hideyuki Okano

**Affiliations:** 1 Department of Physiology, Keio University School of Medicine, Shinjuku, Tokyo, Japan; 2 Division of Neurosurgery, Keio University School of Medicine, Shinjuku, Tokyo, Japan; 3 Pathology and Laboratory Medicine, David Geffen UCLA School of Medicine, Los Angeles, California, United States of America; 4 Division of Gene regulation, Institute for Advanced Medical Research, Keio University School of Medicine, Shinjuku, Tokyo, Japan; University of Chicago, United States of America

## Abstract

Musashi1 (MSI1) is an RNA-binding protein that plays critical roles in nervous-system development and stem-cell self-renewal. Here, we examined its role in the progression of glioma. Short hairpin RNA (shRNA)-based *MSI1*-knock down (KD) in glioblastoma and medulloblastoma cells resulted in a significantly lower number of self renewing colony on day 30 (a 65% reduction), compared with non-silencing shRNA-treated control cells, indicative of an inhibitory effect of *MSI1*-KD on tumor cell growth and survival. Immunocytochemical staining of the *MSI1*-KD glioblastoma cells indicated that they ectopically expressed metaphase markers. In addition, a 2.2-fold increase in the number of *MSI1-*KD cells in the G2/M phase was observed. Thus, *MSI1*-KD caused the prolongation of mitosis and reduced the cell survival, although the expression of activated Caspase-3 was unaltered. We further showed that *MSI1*-KD glioblastoma cells xenografted into the brains of NOD/SCID mice formed tumors that were 96.6% smaller, as measured by a bioluminescence imaging system (BLI), than non-KD cells, and the host survival was longer (49.3±6.1 days vs. 33.6±3.6 days; *P*<0.01). These findings and other cell biological analyses suggested that the reduction of MSI1 in glioma cells prolonged the cell cycle by inducing the accumulation of Cyclin B1. Furthermore, *MSI1*-KD reduced the activities of the Notch and PI_3_ kinase-Akt signaling pathways, through the up-regulation of Numb and PTEN, respectively. Exposure of glioma cells to chemical inhibitors of these pathways reduced the number of spheres and living cells, as did *MSI1*-KD. These results suggest that MSI1 increases the growth and/or survival of certain types of glioma cells by promoting the activation of both Notch and PI_3_ kinase/Akt signaling.

## Introduction

In recent years, substantial research has elucidated the involvement of cancer stem cells in brain tumors [1]. The concept of cancer stem cells originated from observations that not all cancer cells are equal [2]. Brain tumor tissue, for example, which includes both rapidly proliferating cells and post-mitotic differentiated cells, is hypothesized to be generated from the progeny of cancer stem/progenitor cells. Although cancer stem cells are still hypothetical, they are considered a new clinical target for cancer treatments. Thus, many researchers have been trying to distinguish and identify cell surface markers for cancer stem cells, although the validity of these markers is still extensively debated. In addition, cytoplasmic factors in proliferative cell fractions have been investigated as selective markers of cancer stem cells. Among these, the RNA-binding protein Musashi1 (MSI1) has been identified as a candidate marker of cancer stem cells for glioblastoma [3], [4].

The Musashi (Msi) family of RNA-binding proteins is evolutionarily conserved [5], [6]. Msi was originally identified in *Drosophila melanogaster* as a regulator of asymmetric cell division [7], [8], and its mouse counterpart is expressed in stem and progenitor cells in various tissues [6]. Two members of this family, MSI1 and Musashi2 (MSI2), have been identified in mammals [5], [9], [10]. MSI1 is a marker of neural stem progenitor cells (NS/PCs), and functions in stem-cell maintenance in the nervous system and other tissues [11], [12]. We previously demonstrated that MSI1 activates the Notch signaling pathway by translational repression of the mRNA for the Numb protein [13], a negative regulator of the Notch-signaling pathway [14], [15]. The mechanism of MSI1-mediated translational repression was recently clarified in detail: MSI1 competitively inhibits the interaction between eukaryotic translation initiation factor 4G (eIF4G) and poly(A)-binding protein (PABP), thereby blocking translation [16]. Through translational regulation, MSI1 supports the maintenance of the renewal ability of NS/PCs [6]. In addition, it was recently shown that an MSI family molecule suppresses the translation of Numb in chronic myelogenous leukemia (CML) cells [17]. MSI1 is expressed in NS/PCs in the developing embryonic and mature adult brain [11], [12]. In addition, many studies have shown that MSI1 is up-regulated in tumors such as medulloblastoma [18], [19], glioma [3], [4], astrocytoma [20], retinoblastoma [21], and colorectal adenoma [22]. Correlations have been established between the expression level of MSI1 and the grade of malignancy, the cells' proliferative activity [4], and their immaturity, in human glioma [3].

In the present study, to gain insight into the mechanism by which MSI1 contributes to tumor-cell generation or maintenance, we analyzed MSI1's functions in malignant glioblastoma. Furthermore, the contribution of MSI1 to cancer-cell progression and its usefulness as a potential target for cancer therapy have not been examined in animal models *in vivo*. A few reports have been published on the role of *MSI1-*knockdown (KD) in suppressing tumor-cell proliferation in a medulloblastoma cell line [23] and in adenocarcinoma xenografts in nude mice [24]. However, there are no studies clarifying the detailed role of MSI in the tumor growth of glioblastoma, which is the most malignant form of glioma. Hence, here we examined the role of MSI1 in the abnormal growth of glioma cells. Our results show that MSI1 enhance the growth and/or survival of glioma cells by inducing the Notch and PI_3_K/Akt signaling pathways, through post-transcriptional regulatory mechanisms.

## Results

### MSI1 expression in glioblastoma cell lines and low-passage cells obtained from glioblastoma specimens

**Figure 1:**
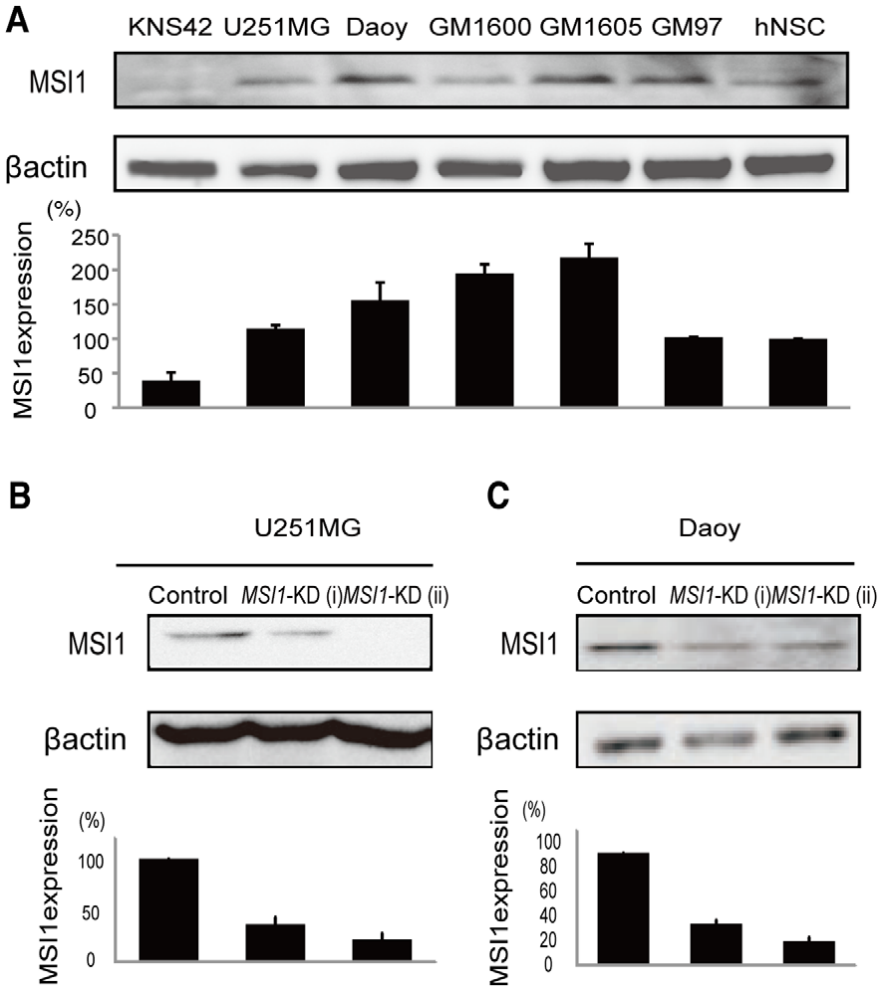
MSI1 expression in glioblastoma cell lines, medulloblastoma cell lines, and low-passage cells from glioblastoma patients. (A) Immunoblots showing the MSI1 expression in human glioblastoma cell lines (U251MG, and KNS42), low-passage glioblastoma cells obtained from patients GM97, GM1600, GM1605, and a human medulloblastoma cell line (Daoy). (B and C) The selective depletion of MSI1 protein by treatment with shRNAs in U251MG cells (B) and Daoy cells (C). β-actin was used as a loading control in all the experiments.

We examined various glioma cells for MSI1 protein expression by immunoblotting, and found it in human glioblastoma cell lines (KNS42 and U251MG), low-passage glioblastoma cell lines obtained from patients (GM1600, GM1605, and GM97), and a human medulloblastoma cell line (Daoy) (Fig. 1A; compare with the level in hNSCs). Each cell line and the primary cells from glioblastoma patients expressed the MSI1 protein at different levels, even though there were no differences in the patients' poor prognosis. The MSI1 protein expression was greater in cancer cells than in hNSCs, except for the KNS42 glioblastoma line. Lentivirus-mediated transduction of two separate anti-*MSI1* shRNAs (*MSI1-*KD (i) and *MSI1-*KD (ii)) selectively depleted the production of MSI1 protein in the U251MG line (Fig. 1B), to 43% and 27%, respectively, and in the Daoy cells (Fig. 1C), to 36% and 21%, respectively, compared to the level in control cells transduced with non-silencing shRNA. The shRNA target sequences of *MSI1* KD (i) and *MSI1* KD (ii) are shown in materials and methods.

### Effect of *MSI1*-KD on glioma cell colony formation

**Figure 2:**
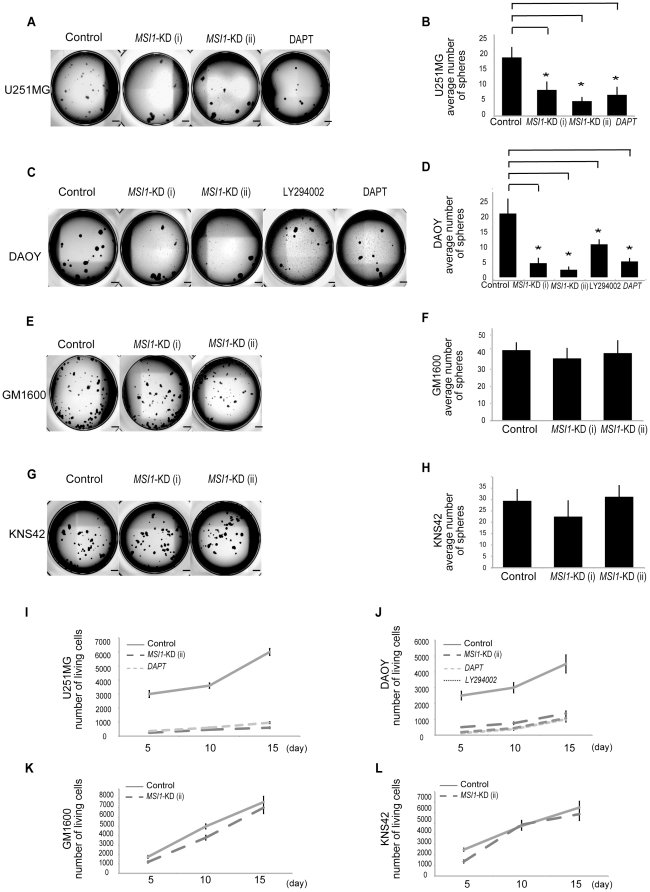
Colony-forming cell assay and effects of *MSI1*-KD. (A,B,C and D) *MSI1* shRNA decreased the number of colony-forming cells. (A) Representative images of the secondary colonies formed by the control shRNA-treated and *MSI1-*KD U251MG cells. (Bar = 500 µm). The number of spheres was reduced in U251 cells exposed to 10 µM DAPT. (B) On Day 30, secondary spheres larger than 100 µm in diameter were counted. U251MG glioblastoma cells transduced with *MSI1* shRNA and cells treated with 10 µM DAPT (γ-secretase inhibitor) generated significantly fewer spheroid cell colonies than cells transduced with control shRNA. Error bars represent SEM, *P<0.01(C) Representative images of the secondary colonies obtained in cells transduced with control shRNA and in *MSI1-*KD PTEN-intact Daoy cells exposed to 10 µM LY294002 (PI_3_ kinase inhibitor) and DAPT. (D) On Day 30, secondary spheres larger than 100 µm in diameter were counted. PTEN-intact Daoy cells transduced with *MSI1* shRNA and cells exposed to LY294002 and DAPT generated significantly fewer spheroid colonies than cells transduced with control shRNA. Error bars represent SEM, *P<0.01(E and G). Representative images of the secondary colonies formed by cells expressing low endogenous MSI1. GM1600 (Fig. 2E) and KNS42 (Fig. 2G) transduced with control shRNA and *MSI1* shRNA (F and H). On day 30, secondary spheres larger than 100 µm in diameter were counted. There was no significant difference between control shRNA and *MSI1* shRNA in GM1600 (Fig. 2F) or KNS42 (Fig. 2H). (I, J, and K) *MSI1*-KD inhibits colony formation. Proliferation was assessed in glioblastoma and medulloblastoma cell lines (cell density of 2×10^3^ cells/well) were assessed for cell proliferation by cell counting. The total cell count in the *MSI1*-KD groups on day 15 was reduced by 83%, and 55% in the (I) U251MG and (J) Daoy cells, respectively, as compared to the control groups.

To evaluate the effect of *MSI1*-KD on tumor cell colony formation *in vitro*, we performed methylcellulose gel colony-forming assays in the presence of EGF and FGF2. Under these conditions, the cells form spherical colonies. The number of colony (at a starting density of 2000 cells per well and counting colonies larger than 100 µm), which indicated progenitor cell proliferation, showed a reduction of 65%, 84%, and 58% in the spheres generated from *MSI1*-KD U251MG, Daoy, and GM1605 cells, respectively. By contrast, in GM1600 (Fig. 2E,F) and KNS42 (Fig. 2.G,H) cells, which have lower endogenous MSI1 expression, the number of colonies was not reduced by *MSI1* shRNA compared to control shRNA. Next, primary spheres were dissociated into single cells and further cultured in methylcellulose-containing medium, resulting in the reformation of spheres (secondary spheres). These secondary spheres were used in all experiments. *MSI1*-KD reduced the number of spheres compared to control shRNA in U251MG (Fig. 2A) and Daoy cells (Fig. 2C). The spheres were then dissociated and the total cell number was counted. On day 15, the total cell number in *MSI1*-KD spheres was reduced by 83%, 55% and 39% of control in U251MG (Fig. 2I), Daoy (Fig. 2J) and GM1605 (Fig. S3) cells, respectively; however no decrease in cell number was observed in GM1600 (Fig. 2K) and KNS42 (Fig. 2L) cells, suggesting that MSI1 does not contribute to proliferation in these cells. Sphere number and cell survival were decreased in U251MG cells exposed to DAPT (γ-secretase inhibitor) and in Daoy cells exposed to LY294002 (PI_3_ kinase inhibitor) and DAPT. These results suggest that MSI1 regulates both the Notch pathway and the PI_3_ kinase/Akt pathway in glioma cells. This is consistent with the observation that cell proliferation was repressed in *MSI1-*KD spheres compared to control spheres, and suggests that *MSI1*-KD may disorder the cell cycle in U251MG and Daoy.

### *MSI1*-KD inhibits the growth of glioblastoma xenografts

To assess the effect of *MSI1-*KD on tumor growth, U251MG or Daoy cells, treated with *MSI1*-KD (ii) or control shRNA, were transplanted into the right striatum of NOD-SCID mice. The transplanted cells were transfected with a bicistronic reporter gene encoding a luminescent protein (CBRluc) and a fluorescent protein (Venus) separated by an IRES. The tumor growth was monitored in live animals by bioluminescence imaging (BLI) using the IVIS system® (Caliper Life Sciences, Hopkinton, MA), with which the grafted cells could be identified by their fluorescent Venus and bioluminescent Luciferase signals. The results of the experiments using U251MG and Daoy cells are shown in Fig. 2, respectively. Treatment of U251MG cells with *MSI1-*KD resulted in a 96.6% reduction in BLI on Day 28 compared to the control shRNA group (Fig. 2A and B), and the host survival time in the *MSI1*-KD group (49.3±6.1 days) was significantly longer than that in the shRNA control group (33.6±3.6 days; *P*<0.01) (Fig. 2C). In Daoy cells, *MSI1-*KD cells resulted in a 97.3% reduction in BLI on Day 28 compared to the control shRNA group, the survival time of the *MSI1*-KD group (45.5±5.4 days) was significantly longer than that of the shRNA control group (34.0±4.1 days; *P*<0.01)(Fig. 2D and E). The successful transplantation of the control U251MG cells (Fig. 2F-a) and of *MSI1-*KD U251MG cells (Fig. 2F-b) into the mouse brain was confirmed by hematoxylin and eosin staining. However, unexpectedly, the number of positive cells for phosphohistone H3 (Fig. 2G-b, d) in the *MSI1-*KD-cell-transplanted mice was much larger than the number in the controls (Fig. 2G-a, c), indicating that the peri-metaphase (M-phase) cell-cycle regulation might be abnormal in the *MSI1-*KD cells. Taken together, these results suggest that the downregulation of MSI1 in the tumors led to a reduction in tumor tissue mass and to disordered cell proliferation and cell-cycle regulation.

### Treatment with *MSI1*-KD induces mitosis and G2/M arrest in spheres of glioblastoma cells

**Figure 3:**
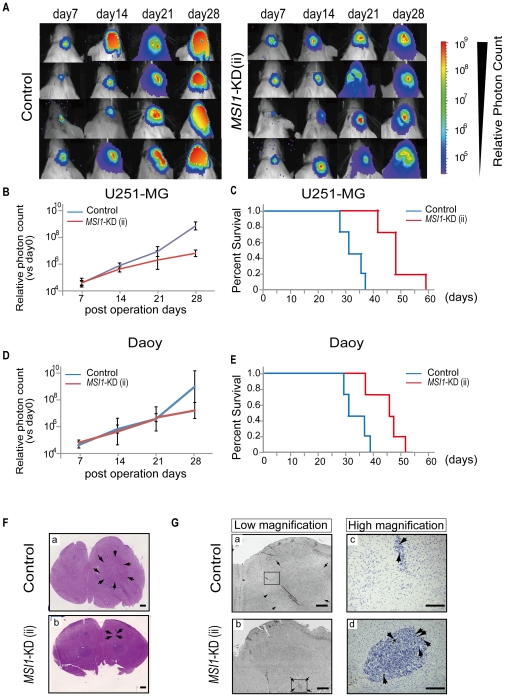
Xenografts expressing *MSI1*-KD showed reduced BLI and longer survival. Briefly, 1×10^4^ human glioblastoma cells were transplanted into the right striatum of NOD/SCID mice (n = 4), and tumor growth was monitored in live animals. The *MSI1*-KD group exhibited better survival than the control group (n = 4, ^*^P<0.05). (A) Representative BLI images of mice receiving implants of *MSI1* shRNA lentivirus-transfected U251MG cells on day 28. (B&D) Relative photon count of BLI compared with that day0 was shown. The tumor average photon count in *MSI1*-KD group was inferior to that in control groups in U251MG cells (Fig. 3B) and in Daoy cells (Fig. 3D) (C) Kaplan-Meier survival curves for the control and *MSI1*-KD mice in U251MG cells (^*^P<0.05). The survival time in the *MSI1*-KD group (49.3±6.1 days) was significantly longer than that in the shRNA control group in U251-MG cells (33.6±3.6 days; *P*<0.01). (E) Kaplan-Meier survival curves for the control and *MSI1*-KD mice are shown in Daoy cells (^*^P<0.05). The survival time of the *MSI1*-KD group (45.5±5.4 days) was significantly longer than that of the shRNA control group in Daoy cells (34.0±4.1 days; *P*<0.01). (F) Mouse brain transplanted with control cells (F-a) and *MSI1*-KD cells (F-b) was stained for hematoxylin and eosin (Bar = 1 mm). (G) Brain transplanted with control cells [G-(a, c)] and *MSI1-*KD cells [G-(b, d)] was immunostained for PH3 [(a, b) Bar = 800 µm (c, d) Bar = 200 µm]. In G-(a, b), surrounding arrows show the transplanted tumor area. G-(c, d) indicated the high magnification images of the squared region of G-(a, b), respectively.

**Figure 4:**
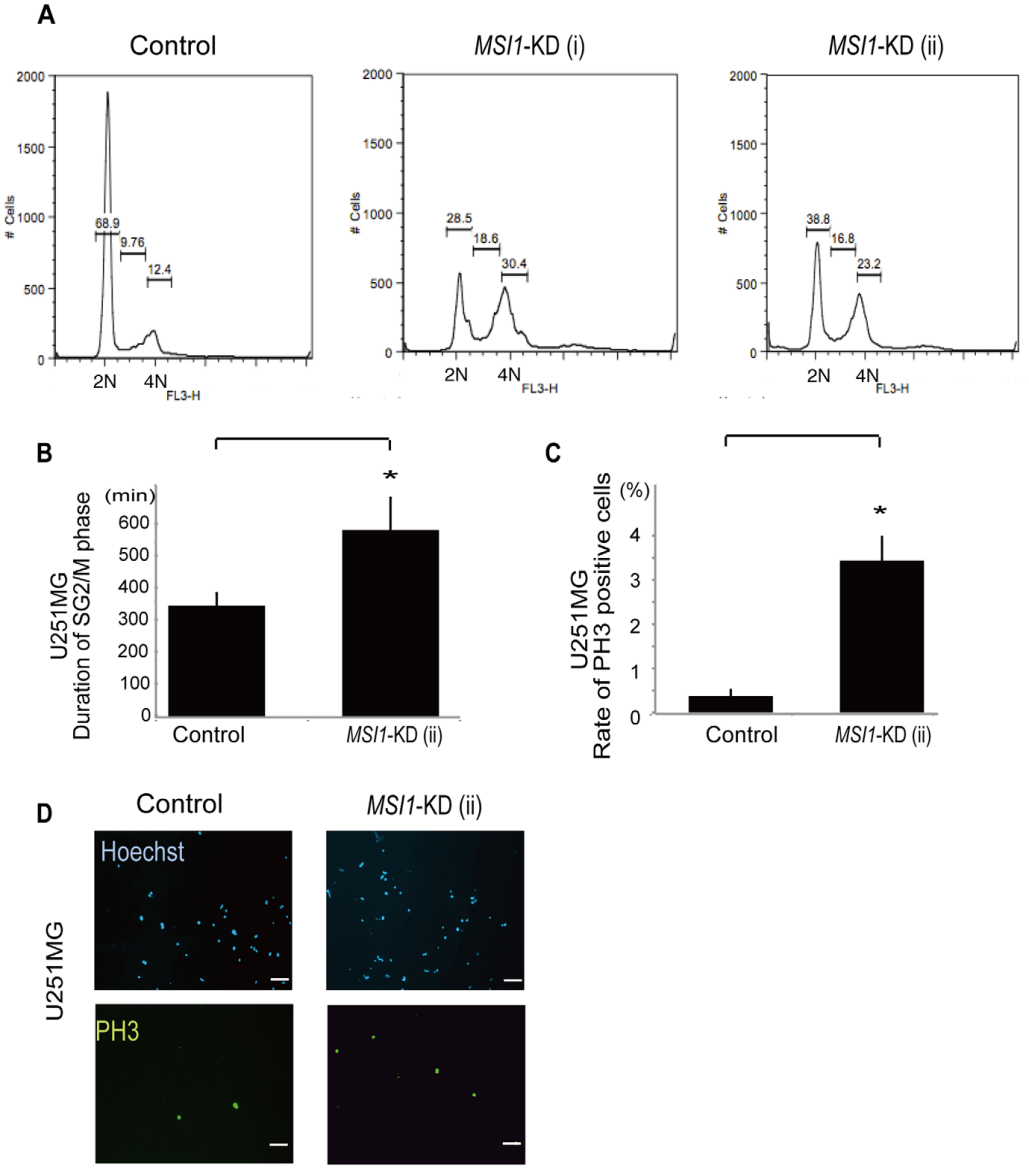
Knockdown of MSI1 induces G2/M arrest. *MSI1*-KD cells were analyzed by fluorescence-activated cell sorting. A representative cell-cycle profile for each treatment is indicated. 2N represents the G0–G1 phase and 4N represents the G2/M phase. An increased number of *MSI1* KD-positive cells accumulated in the G2/M phase. (B) Time-lapse microscopy using a fluorescent ubiquitination-based cell-cycle indicator (Fucci) probe system showed that *MSI1*-KD cells retained the green fluorescence for a much longer time (4 days). Error bars represent SEM, *P<0.01.The spheres were dissociated, and the dissociated cells were fixed to coverslips and stained for PH3 in U251MG cells (C and D). Cell counts are plotted as bar graphs. An average of 25 high-power fields was counted. Error bars represent SEM, *P<0.01. (D) Representative images of control cells and *MSI1*-KD cells stained for PH3 and Hoechst. There were more PH3-positive *MSI1-*KD cells than control cells. (Bar = 500 µm).

The decrease in the MSI1 protein level in brain tumor cells led to a reduction in the transplanted tumor mass in the host mouse brain (Fig. 2) and a repression of cell proliferation (Fig. 3). We therefore surmised that MSI1 regulates both the cell-cycle and cell survival. To analyze the effects of *MSI1-*KD on the cell cycle, *MSI1*-KD U251MG cells were analyzed for cell-cycle distribution by flow-cytometry. Compared to control cells, a higher number of *MSI1*-K*D* U251MG cells accumulated in the G2/M phase, and significantly fewer cells in the G0–G1 and S1 phases were detected. These results clearly demonstrate that the knockdown of *MSI1* in U251MG cells resulted in changes in the cell-cycle phase distribution (Fig. 4A).

To further analyze the cell-cycle status of *MSI1*-KD U251MG cells, we used a ubiquitination-based fluorescent cell-cycle indicator (Fucci) probe-based system, in which transfected cells show green fluorescence during the G2/M phase of the cell cycle [25]. The *MSI1*-KD cells retained the green fluorescence for a much longer time (4 days), as observed by time-lapse microscopy (Fig. 4B), than the control U251MG cells, suggesting a clear relationship between MSI1 and the cell cycle.

The cell colonies were then dissociated, fixed, and immunostained for phosphohistone H3 (PH3) to assess the cells' mitotic status. Both *MSI1*-KD U251MG cells and control U251MG cells stained positive for PH3, a marker for mitosis. Compared to the control cells, however, the *MSI1*-KD-transduced U251MG cells (Fig. 4C and 4D) showed a significant, 10-fold increase in PH3-positive cells. In GM1605 cells, the *MSI1-*KD-transduced cells demonstrated an increase of 2.8-fold (Fig. S1). Taken together, these results indicate that MSI1 is involved in cell-cycle control, especially at the M-phase, in brain tumor cells.

### *MSI1*-KD increases Caspase-3-independent cell death through a mechanism other than cellular senescence or apoptosis

**Figure 5:**
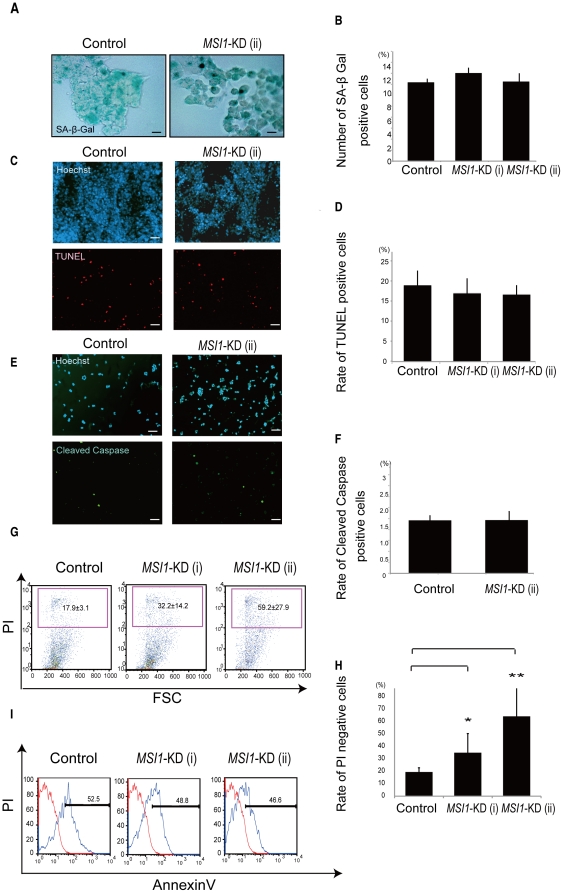
*MSI1*-KD cells may undergo non-apoptotic cell death. (A and B) SA-β-Gal staining was performed to assess the senescence in controls and *MSI1-*KD cells. (A) Representative images of control cells and *MSI1*-KD cells stained for SA-β-Gal (Bar = 200 µm). (C, D, E, and F) Colonies were dissociated, and the cells were fixed to coverslips and stained for TUNEL in U251 MG cells (C and D), and for cleaved Caspase-3 in U251MG cells (E and F). Cell counts are plotted as bar graphs. An average of 25 high power fields was counted. (G and H) The number of PI-positive cells (indicating dead cells) was greater in the *MSI1*-KD cell population than in the control cell population. Error bars represent SEM. *P<0.01.**P<0.01. (I) The number of annexinV-positive in the *MSI1*-KD cells (indicating apoptotic cells) was not different from that in the control cell population.

Apoptosis, senescence, or the alteration of cell survival was possible reason for the cell-number reduction in the *MSI1*-KD tumor cell colonies. To determine whether any of these processes was involved, we performed immunocytochemical experiments. To evaluate the state of senescence, the *MSI1*-KD cells were stained for senescence-activated β-Galactosidase (SA-β-Gal) [26]. There was no significant difference between the control and *MSI1*-KD groups on day 30 (Fig. 5A and B). Next, the *MSI1*-KD cells were dissociated, fixed, and immunostained for TUNEL and anti-activated Caspase-3 to examine apoptotic targets. There was no significant difference in the number of TUNEL- (Fig. 5C and 5D) or activated Caspase-3-positive cells (Fig. 5E and 5F) between the *MSI1*-KD U251MG cells and control cells. Similar results were obtained using *MSI-*KD GM1605 cells (Fig. S2). Further flow-cytometric population analysis showed that the number of annexin V positive cells in the *MSI1*-KD group were the same cells compared with control group (Fig. 5I), inducing the conclusion that there were no difference in apoptotic cells between *MSI1*-KD U251MG cells and control cells. However, flow-cytometric population analysis revealed that the number of cells that incorporated propidium-iodide (PI) was higher for the *MSI1*-KD cells compared with control cells (Fig. 5G and 5H), indicating that there were more dead *MSI1*-KD U251MG cells than control cells. Therefore, *MSI1*-KD appeared to induce cell death simultaneously with the occurrence of cell-cycle dysregulation. However, no increase in cleaved-Caspase-3-positive cells and in TUNEL positive cells were observed in the *MSI1*-KD tumor cells. Collectively, these results suggested that the *MSI1*-KD-induced reduction in colony-forming cells in the glioblastoma cell line occurs through cell death, but via a pathway other than apoptosis, possibly necrosis.

### *MSI1-*KD alters the expression of several cell cycle-related markers and PTEN-PI_3_ kinase/Akt pathway-related molecules in some glioma cells

**Figure 6:**
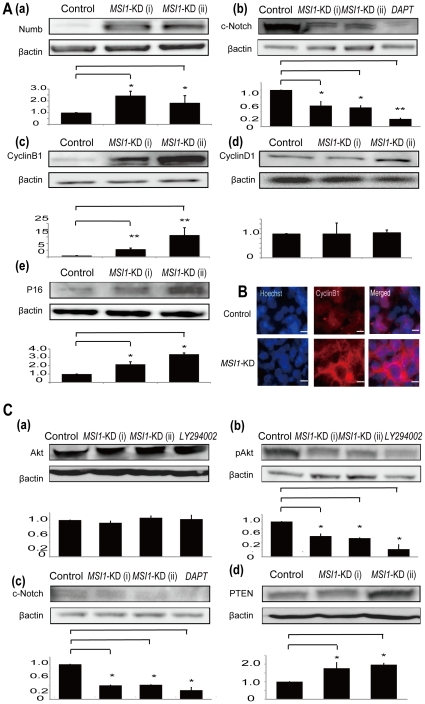
*MSI1-*KD changed the expression level of various genes. (A) Immunoblots showing the expression of various cell-cycle markers and downstream genes of MSI1 in control and *MSI1*-KD in U251MG cell lines. *MSI1*-KD resulted in the up-regulation of Numb, Cyclin B1, and P16, the downregulation of cleaved Notch, and no change in CyclinD1. (B) Immunocytochemistry showed that Cyclin B1 was potently expressed in *MSI1*-KD cells compared with control cells. (Bar = 200 µm). (C) In PTEN-intact Daoy cells, the immunoblot analysis showed that *MSI1-KD* led to the up-regulation of PTEN, and down-regulation of cleaved Notch and p-Akt with no change in total Akt level. Error bars represent SEM (*P<0.01).

To clarify the events occurring in the *MSI1*-KD cells, we analyzed whether there were differences in the expression levels of various proteins between *MSI1*-KD cells and control shRNA-transduced U251MG (Fig. 6A) and Daoy cells (Fig. 6C). MSI1 activates Notch signaling thorough the MSI1-Numb-Notch pathway [6], [27], and the excessive translational repression of Numb by the constitutive overexpression of MSI promotes the development and progression of CML blast crisis [17]. Therefore, we examined the *MSI1*-KD mediated alteration of the Numb protein level and Notch activity, as represented by the cleaved-Notch level in glioma cells. Furthermore, because we found that MSI1 reduction was accompanied by cell-cycle prolongation, as shown in Figs. 3 and 4, and apparently regulated the cell-cycle, especially at the M-phase, we examined the changes in cell-cycle-related markers caused by *MSI1*-KD. Cyclin B1 is involved in mitosis, and Cyclin D1 is involved in the G1/S transition; PTEN are suppressors of Akt pathways. An increased expression of the *p16* gene as organisms age reduces the proliferation of stem cells [28]. Since our results shows MSI1 was involved in glioma survival, we surmised MSI1 was related to the regulation of PTEN-PI_3_ kinase/Akt pathway. Our results showed that *MSI1*-KD induced the up-regulation of Numb, Cyclin B1, p16, and reduced the level of cleaved Notch in U251MG cells. Furgthermore, *MSI1-*KD up-regulated PTEN, and down-regulated the level of cleaved Notch and Akt phosphorylation at theonine 308 (Phospho-Akt (Thr308)) in PTEN-intact Daoy cells (Fig. 6C). There was no change in the expression of Cyclin D1 compared to control U251MG cells (Fig. 6A) and no change in the expression of Akt in Daoy cells (Fig. 6C). Cyclin B1 was more strongly expressed in *MSI1*-KD cells than in control cells by immunocytochemistry (Fig. 6B). In the *MSI1* KD cells, Cyclin B1 was located in the cytoplasm, indicating the cells were in early mitosis. In M-phase, the nucleus has split in two and is not entirely stained by Cyclin B1 (Fig. 6B). Taken together, these results show that a decrease in MSI1 led to decreases in both the Notch pathway in U251MG cells and the PI_3_ kinase-Akt signaling activity in Daoy cells. Furthermore, Cyclin B1 was up-regulated, which is consistent with the prolongation of metaphase observed upon *MSI1*-KD.

## Discussion

Here, by knocking down *MSI1* in glioma cells, we found that *MSI1* may promote cell proliferation and survival, and its loss can be detrimental. *MSI1-*KD in medulloblastoma Daoy cells induces apoptosis and mitotic catastrophe, suggesting a potential mechanism for its role in tumorigenesis [23]. *MSI1-*KD in adenocarcinoma cells results in tumor growth arrest in xenografts, reduced cancer cell proliferation, and increased apoptosis [24]. MSI1 acts as a key determinant of the mammary lineage through its inability to coordinate cell-cycle entry and activate the Notch and Wnt pathways [24], [27]. In human breast cancer patients, MSI1 is a negative prognostic indicator of patient survival, and is indicative of tumor cells with stem cell-like characteristics [29]. These previous reports and our current findings showed that *MSI1*-KD reduces tumor-cell survival and tumor-xenograft growth, and support its possible identification as a novel target for glioblastoma therapy.

In this study, using an *in vitro* approach, we showed that MSI1 is highly expressed in various glioblastoma cell lines (Fig. 1). *MSI1*-KD treated cells generated a reduced number of glioma spheres (Fig. 2) and stained positive for M-phase markers (Fig. 4). In addition, a tumor-growth assay *in vivo* using NOD-SCID mice demonstrated that xenografts expressing *MSI1*-KD resulted in a smaller tumor size (as seen by a marked decrease in BLI) and longer mouse survival compared with their counterparts treated with control shRNA-expressing xenografts (Fig. 3). These results were consistent with previous studies indicating that high MSI1 expression is associated with a poor prognosis in glioma [3], [4].

Furthermore, in glioblastoma cells, we found that *MSI1*-KD led to decreased Notch signaling activity, as represented by the level of cleaved Notch, and to the accumulation of Numb. Our group previously reported that MSI1 represses Numb translation and consequently augments the Notch signaling activity [27]. Other groups have shown that MSI1 up-regulates the Notch signaling activity in medulloblastoma [18], colon cancer [24], mammary progenitor cells [29], and endometrial carcinoma [30]. The nuclear translocation of Notch1, which results in the activation of its downstream pathway, may contribute to the induction of tumorigenesis by increasing proliferation and inhibiting apoptosis [2], [31].

**Figure 7:**
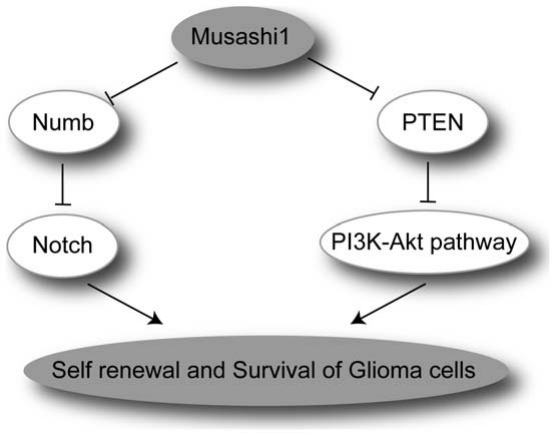
Current model for the involvement of Musashi-1 in glioma development. The elevated level of the RNA-binding protein Musashi-1 leads to the down-regulation of Numb and PTEN (Imai et al., unpublished), resulting in increased activity of the Notch and PI3K/Akt-pathways, respectively. These events could lead to the enhanced self-renewal and survival of glioma cells and subsequent glioma development. In addition to these mechanisms, the downregulation of Cyclin B1 expression and prolongation of the cell cycle could be involved in Musashi1-mediated glioma development.

A recent report showed that the MSI2-mediated downregulation of Numb significantly impairs the development and propagation of CML blast crisis *in vitro* and *in vivo* [17]. Indeed, disorganization of the Notch signaling balance by breakdown of the Musashi-Numb axis is directly linked to the malignancy in the CML blast crisis [32]. Consequently, modulation of the Musashi-Numb-Notch pathway influences the tumorigenicity of various tumors including glioblastoma, as described in this study. *MSI1*-KD probably leads the glioblastoma cells to mitotic catastrophe, resulting in cell death by the accumulation of Numb protein and subsequent inhibition of the Notch signaling pathway. (Fig. 7)

The reduction of MSI1 in glioblastoma cells caused other abnormalities in cell-cycle regulation. *MSI1*-KD increased the number of PH3-positive cells (Fig. 4C, Fig. S1), and the cell-cycle analysis using PI revealed that the number of *MSI1*-KD cells in the G2/M phase increased (Fig. 4A). Time-lapse assays using Fucci demonstrated that *MSI1*-KD sphere cells stayed longer in the S/G2/M phase of the cell cycle than cells in control spheres (Fig. 4B). There was a significant decrease in the number of *MSI1*-KD cells accumulating in the G0–G1 phase, supporting the previous finding that MSI1 promotes G1/S transition through the activation of both the Notch and Wnt signaling pathways [29]. Immunoblot analysis revealed that *MSI1*-KD in glioblastoma cells resulted in the accumulation of Cyclin B1 due to prolongation of the M-phase (Fig. 6). Interestingly, MacNicol's group found that Msi1 activates the cytoplasmic polyadenylation of *c-Mos*, *Cyclin B1*, and *Cyclin B4* mRNA in *Xenopus* oocytes [33], [34]. In the *Xenopus* oocyte, cytoplasmic polyadenylation is thought to be linked with translation activation [35]. The *c-Mos* gene is related to meiotic cell-cycle progression. However, in our glioblastoma cells, Cyclin B1 was probably appropriately down-regulated by MSI1 through an indirect or direct pathway. Why MSI1 has different functions in various contexts is an interesting question for future study.

There was no difference in the expression of the apoptotic marker activated Caspase-3 (Fig. 5E, 5F, and Fig. S2) or in TUNEL staining (Fig. 5C and 5D) in *MSI1*-KD-expressing cells, and the number of annexinV-positive cells was the same as the shRNA-transduced control cells (Fig. 5I), but the number of PI-positive dead cells increased (Fig. 5G and 5H). In the present study, the decrease in MSI1 in glioblastoma cells led to cell growth retardation and a defect in non-apoptotic cell survival, accompanied by a cell-cycle abnormality, which resulted in an ectopic accumulation of Cyclin B1. These findings showed that the decrease in cell growth was caused by prolongation of the cell cycle, especially by a prolonged M-phase, and an increase in cell death by a Caspase-3-independent mechanism. Previously, inappropriate increases in nuclear Cyclin B1 have been found in mitotic catastrophe in colorectal adenocarcinomas, nasopharyngeal carcinoma, and colon cancer [36], [37]. It has been suggested that an elevated level of Cyclin B1 or premature activation and nuclear entry of CDK1/Cyclin B1 may be sufficient to induce mitosis before the completion of DNA replication, thus causing cell death during mitosis [38]. An inhibition of Notch signaling results in prolonged G2-M cell-cycle arrest before the induction of apoptosis in Kaposi's sarcoma, and leads to mitotic catastrophe, in which the destruction of nuclear structures is observed [39].

These phenomena correspond to mitotic catastrophe. The initial step in mitotic catastrophe is evidence of DNA damage in a cell that is undergoing mitosis [39]. A hallmark of mitotic catastrophe is the entry of cells into mitosis despite the presence of damaged DNA, resulting in the activation of cell death [40], [41]. These facts led us to hypothesize that Notch inhibition in glioblastoma cells results in mitotic catastrophe, which is finally resolved in prolongation of the cell cycle and delayed cell growth. Therefore, *MSI1*-KD probably leads the glioblastoma cells to undergo mitotic catastrophe, resulting in cell death caused by the accumulations of Numb and Cyclin B1 proteins and by the inhibition of Notch.

It is known that glioblastomas often contain mutations and deletions in the tumor suppressor gene PTEN, whose alteration affects the phosphatidyl-inositol-3 kinase (PI_3_K)/Akt pathway [42], [43]. In *MSI1*-KD Daoy cells, the expression of PTEN was increased (Fig. 6C). Recently, in an MSI1-RNA co-immunoprecipitation experiment, we found that PTEN mRNA is a putative downstream target of MSI1 in mouse neural stem cells (Imai et al., *unpublished* results). In the present study we observed an increase in the PTEN protein level, a decrease in the phosphorylation level of Akt (at Thr308), and no alteration in the Akt protein level in *MSI1-KD* cells. Accordingly, it is possible that PTEN is down-regulated by MSI1 in glioma cells. The number of spheres and living cells was reduced in PTEN-intact Daoy cells upon exposure to LY294002 (PI_3_ kinase inhibitor) and DAPT (γ-secretase inhibitor), a phenotype also observed upon *MSI1-*KD (Fig. 2).

We hypothesize that competence for cell survival may be regulated by the MSI1-PTEN-PI_3_ kinase/Akt pathway in glioma cells. PTEN negatively regulates neural stem cell self-renewal by modulating G0–G1 cell-cycle entry [44]. Notably, in hematopoietic stem cells, deletion of the *Pten* gene results in the generation of leukemic stem cells but the depletion of normal hematopoietic stem cells [45]. Taking these reports and our data together, our current model is that MSI1 plays a role in glioma development by enhancing the self-renewal and survival of glioma cells through the MSI1-Numb-Notch pathway and the MSI1-PTEN-PI_3_ kinase-Akt pathway (Fig. 7).

Although the MSI1-binding targets in HEK293 have been described [46], they are not known in glioblastoma cells, and our future research will be directed at identifying the downstream pathway of MSI1 through microarray analysis and proteomic profiling in glioblastoma cells. Knowledge about the downstream target genes of MSI1 will shed light on the mechanism by which these proteins promote cancer cell progression. Based on our *in vitro* and *in vivo* findings, we propose that tumor stem cells treated with *MSI1*-KD lose their self-renewal ability and undergo a non-apoptotic programmed cell death, which may explain the difference in tumor growth between the control and *MSI1*-KD groups. However, results showed that the level of MSI1 expression did not always correlate with malignancy: considering the fact that gliomas are highly variable in genetic changes/mutations and cellular origin, it is conceivable that both *MSI1*-dependent and -independent mechanisms are involved in the regulation of malignancy in glioma.

## Materials and Methods

This study was approved by our local institutional ethical review board,“KEIO university ethical committee”. The establishment of glioblastoma cell lines GM97, GM1600, and GM1605 was specifically approved by the institutional review board of the university of California at Los Angels. This study was performed in accordance with the ethical standards laid down in the 1964 Declaration of Helsinki. All the animal experiments were conducted according to the Guidelines for the Care and Use of Laboratory Animals of the Keio University School of Medicine. The KEIO permit number is #2634 and the UCLA approval number is: UCLA IRB APPROVED PROTOCOL: #10-000344. Written informed consent was obtained from all participants involved in the study.

### Cell culture

Human glioblastoma cell lines U251MG, KNS42, KNS81, KNS60, and SF126 were purchased from Japanese Collection of Research Bioresources (JCRB). Other glioma cell lines, U87MG and T98G were purchased from ATCC. GM97, GM1600, and GM1605 were established from surgically resected specimens from glioblastoma patients at the University of California Los Angeles, in accordance with protocols approved by the University of California Los Angeles Institutional Review Board, as previously described [29], [47]. Briefly, after the tumor was histologically diagnosed as glioblastoma by a board-certified neuropathologist, it was finely minced with a scalpel and resuspended in complete Iscove's modified 20% Dulbecco's medium (Invitrogen) supplemented with 10% fetal bovine serum (FBS) (Omega), 2 mM glutamine, 5 µg/mL each of insulin and transferrin, 5 ng/mL selenium (ITS Culture Supplement; Collaborative Biosciences, Bedford, MA). The GM97, GM1605, and GM1600 cell lines were passaged fewer than 10 times. The T98G, KNS42, KNS81, KNS60, SF126, GM97, GM1605, and GM1600 cell lines were maintained in Dulbecco's modified Eagle's medium (DMEM) (Sigma-Aldrich Chemical Co., St. Louis, MO). The U251MG cell line was maintained in modified Eagle's medium (MEM) (Sigma-Aldrich). Sphere formation was performed as previously described [48]. Cells were cultured in serum-free DMEM HAM/F12 medium (Sigma-Aldrich Chemical Co., St. Louis, MO) supplemented with 1×B27 (Invitrogen), 20 ng/mL epidermal growth factor (EGF; Peprotech Invitrogen), and 20 ng/mL fibroblast growth factor-2 (FGF-2; Peprotech Invitrogen) for immunostaining and western blot experiments. The cultures were incubated at 37°C in a 5% CO_2_ cell culture incubator (Sanyo). Glioblastoma and medulloblastoma cells grown as sphere cultures were collected by gentle centrifugation (800×g, 5 min) and trypsinized with 0.05% TrypLESelect (Invitrogen Corp., Carlsbad, CA) for 5 min by pipetting. Cells were washed twice with phosphate buffered saline (PBS), counted, and seeded at a density of 2000 cells per 200 µL into 96-well ultralow-attachment plates (Corning).

### Immunoblotting

Immunoblot analysis was performed as described previously [16]. Total cell extract (about 30 µg) was fractionated by 10% SDS-PAGE. Proteins were detected using the appropriate primary antibodies and horseradish peroxidase-linked secondary antibodies, and visualized by chemiluminescence (Amersham Biosciences).

### Antibodies used in immunoblot analysis

The following primary antibodies were used for immunoblots: rat anti-MSI1 14H1 [49] or anti-MSI1 14H1 biotinylated (1∶500), rabbit anti-PTEN (1∶500) (Cell Signaling Technology), rabbit anti-p16 (1∶500) (Cell Signaling Technology), mouse anti-p21 (1∶500) (Cell Signaling Technology), rabbit anti phosphorylation at Threonine 308 of Akt (1∶250) (Cell Signaling Technology), rabbit Akt (1∶500) (Cell Signaling Technology), and mouse anti-β-Actin (1∶1000) (Sigma-Aldrich Corp MO). For detection, horseradish peroxidase (HRP)-conjugated anti-rabbit IgG (Jackson ImmunoResearch) (1∶1000), HRP-conjugated anti-rat IgG (Jackson ImmunoResearch) (1∶1000), HRP-conjugated anti-mouse IgG (Jackson ImmunoResearch) (1∶1000), and HRP-conjugated anti-goat IgG (Jackson ImmunoResearch) (1∶1000) were used.

### Colony-forming assay

Primary spheres were collected, incubated in 0.25% trypsin-EDTA for 5 min at 37°C, and triturated until single-cell suspension was obtained. The cells were spun at 800 g for 5 min at 4°C and resuspended in the aforementioned sphere culture medium. The cells were cultured in the above medium with 0.8% methylcellulose (Nacalai Tesque 22224-55) as previously described [50]. The colony-forming cell assay was performed by seeding 3×10^3^ tumor cells/well into 96-well ultralow-attachment plates (Corning) in 200 µL medium. Every four days, 50 µL of the fresh growth medium was added. The number of colonies was counted after 30 days, once the spheres were greater than 100 µm in diameter. The colony (sphere) was observed using fluorescence microscopy (BIOREVO BZ-9000; Keyence). U251MG and DAOY cells were exposed to 10 µM LY294002 (PI_3_ kinase inhibitor, Promeg,WI) and 10 µM DAPT (γ-secretase inhibitor, Sigma-Aldrich Corp., Saint Louis, MO) for 16 days. The culture medium was replaced every 4 days. Sphere formation and cell numbers were assessed. The proteins were collected 24 hours after the last addition of LY294002 and DAPT. For immunocytochemistry, the spheres were collected, incubated in 0.25% trypsin-EDTA for 5 min at 37°C, and triturated until a single-cell suspension was plated in the aforementioned sphere culture medium without serum on poly-D-lysine/laminin (Sigma P7405/Invitrogen 23017-015)-coated 24-well chamber slides (Iwaki 5732-008) and cultured for 2 hours. The samples were observed with a universal fluorescence microscope (Axioplan 2 imaging; Carl Zeiss).

### Flow cytometer-assisted cell sorting

For cell-cycle analysis, dissociated cells (1×10^6^ cells) were incubated in 1 ml of hypotonic propidium iodide (PI) solution (1 µg/ml PI, 0.1% citric acid, 0.2% NP-40, 10 µg/ml RNaseA) [51] for 30 min at 48°C, followed by a 15-min incubation at 37.8°C to digest the RNA. The cells (1×10^7^cells) were suspended in 100 µlHanks' balanced salt solution (HBSS) and incubated on ice for 30 min with fluorescein isothiocyanate (FITC)-conjugated Annexin V (BD Biosciences) to detect apoptotic cells. The cells were washed, resuspended in HBSS containing 1 µg/ml PI, and analyzed by FACS Caliber (BD Biosciences). The effects of cell death were assessed by Annexin V-FITC flow cytometry as previously described [52]. Briefly, the spheres of U251-MG cells were harvested and stained with Annexin V-FITC according to the instructions of the manufacturer. Cell samples were analyzed on a FACS and apoptotic fractions were determined.

### RNA interference experiment

MSI1 expression was reduced using a pLKO lentiviral vector (Sigma) expressing targeting and non-targeting sequences. Concentrated virus harboring the *MSI1* shRNA or a non-silencing shRNA as a negative control was prepared was prepared in 80 µl of PBS. , 20 µl of virus solution was mixed with culture medium to transduce U251MG, Daoy, GM1605, and GM1600 cells. Infected cells were selected by incubation with 2.5 µg/mL puromycin (Sigma-Aldrich) for 10 days. The cells were then harvested and lysed. The efficacy and specificity of the shRNAs were evaluated by immunoblot analysis (Fig. 1).

### Virus production

HEK293FT cells (Invitrogen) were transfected with the lentivirus constructs pCMV-VSV-G-RSV-Rev and pCAG-HIVgp by using Lipofectamine 2000 (Invitrogen) according to the manufacturer's instructions [53]. After incubation for 72 hours, the virus-containing supernatant was collected and centrifuged at 25000 rpm for 90 min at 4°C. Pelleted virus particles were dissolved in 80 ml of PBS.

### RNAi target sequence

*MSI1*-targeting RNAi

5′-CCGGCAAGATGGTGACTCGAACGAACTCGAGTTCGTTCGAGTCACCATCTTGTTTTTG-3′ in the 3′-UTR of *MSI1* mRNA 370 (shown as *MSI1*-KD(i)) and 5′-CCGGCACGTTTGAGAGTGAGGACATCTCGAGATGTCCTCACTCTCAAACGTGTTTTTG-3′ in the 3′-UTR (untranslated region) of *MSI1* mRNA 529 (shown as *MSI1*-KD(ii)) (Oligo ID # V2HS_280120; Open Biosystems, Huntsville, AL).

Non-targeting control vector 5′-CAACAAGATGAAGAGCACCAA-3′ (MISSION Non-Target shRNA Control Vector code# SHC002 Sigma)

### Immunohistochemistry

Animals were anesthetized and transcardially perfused with 4% PFA. The whole brain was removed and postfixed for 8 hr in 4% PFA, soaked overnight in 15% followed by 30% sucrose, and embedded in OCT compound. Coronal sections 14-µm thick were made with a Cryostat (Leica, Wetzlar, Germany) and processed for immunohistochemical analysis.

The tissues were fixed with 4% paraformaldehyde (PFA) in phosphate-buffered saline (PBS) (pH 7.4), followed by cryoprotection with 20% sucrose. Frozen 14-µm-thick tissue sections were prepared using a cryostat (CM3000, Leica). The slides were washed 3 times in PBS, blocked for 1 hour in a buffer containing 10% goat serum in PBS, and incubated overnight at 4°C with rat anti-MSI1 antibody 14H1 [49]. The slides were washed 3 times with PBS, and the antigen was visualized with ABC Vectastain and DAB as substrates (Vector Labs, Burlingame, CA). The slides were counterstained with Harris-modified hematoxylin (Thermo-Fisher, Pittsburgh, PA) and mounted in Permount. The sections were incubated with primary antibodies in TNB blocking buffer (PerkinElmer) at 4°C overnight, and then with fluorescent dye-conjugated secondary antibodies at room temperature for 1 hr. The images were observed by fluorescence microscopy (Axioplan2 Imaging, Carl Zeiss and BZ9000, Keyence Osaka, Japan).

### Antibodies used in immunocytochemistry

For immunocytochemistry, rat anti-Cyclin B1 (1∶500) (Santa Cruz Biotechnology) was used in addition to antibodies used in immunoblot analysis shown above. The secondary antibodies were as follows: Alexa488 conjugated goat anti-mouse IgG (Invitrogen) (1∶1000), Alexa488-conjugated goat anti-rabbit IgG (Invitrogen) (1∶1000), Alexa488-conjugated goat anti-rat IgG (Invitrogen) (1∶1000), Alexa488-conjugated donkey anti-rabbit IgG (Invitrogen) (1∶1000), Alexa488-conjugated donkey anti-rat IgG (Invitrogen) (1∶1000), Alexa555-conjugated goat anti-rat IgG (Invitrogen) (1∶1000), Alexa555-conjugated goat anti-rabbit IgG (Invitrogen) (1∶1000), Alexa555-conjugated donkey anti-goat IgG (Invitrogen) (1∶1000), anti-Hoechst(Sigma-Aldrich) (1∶1000), and Alexa568-conjugated donkey anti-sheep IgG (Molecular Probes) (1∶1000).

### Xenograft transplantation into mouse brain

All the animal experiments were conducted according to the Guidelines for the Care and Use of Laboratory Animals of the Keio University School of Medicine. Mice were anesthetized and the implantation of partially dissociated human glioma spheres (1×10^4^ cells in 3 µl of PBS) into the right striatum was performed stereotactically (2 mm lateral and 1 mm anterior to bregma; depth 3 mm from dura). We used immunodeficient mice (NOD/SCID mice; Charles River, Tokyo, Japan) to avoid rejection of the xenografts. The survival after glioblastoma cell transplantation was evaluated for up to 2 months.

### SA-β-gal staining

Senescence-associated β-galactosidase (SA-β-gal) staining was performed as described previously [26]. Briefly, the control and MSI1-KD spheres were washed in PBS, dissociated, fixed with 2% formamide-0.2% glutaraldehyde in PBS for 5 min at room temperature, and incubated overnight at 37°C in fresh X-Gal solution (1 mg/ml X-Gal, 5 mM potassium ferrocyanide, 5 mM potassium ferricyanide, 150 mM NaCl, and 2 mM MgCl_2_ in 40 mM citric acid/sodium phosphate buffer, pH 6.0). The next day, the cells were rinsed with PBS, and the percentages of SA-β-gal-positive (blue) cells were determined after scoring 100 cells for each sample using a bright-field microscope.

### Statistical analysis

Statistical analyses were performed using Student's t-test and Dunnett's test. Student's t-test was used to compare the control shRNA groups with the *MSI1*-KD groups in the colony-formation assay, immunocytochemistry, and the cell number assay. Values are presented as the mean ± SEM. Significance was accepted at P<0.05

### TUNEL staining

The cells were stained with Hoechst 33258 and then treated with the ApopTag

Red In Situ Apoptosis Detection Kit (Intergen Co. Ltd, NY, USA). The number of positive cells was counted by an IN Cell Analyzer 2000 (GE Healthcare Bioscience).

### Bioluminescence Imaging (BLI)

To monitor the tumor growth in live animals, we used the IVIS system® (Caliper Life Sciences, Hopkinton, MA). The cells used for grafting were transduced with a lentivirus containing the click beetle red Luciferase (CBRluc) coding sequence and a Venus bicistronic reporter gene connected by an internal ribosomal entry site (IRES) (EF1α-CBRluc-IRES-Venus). Specifically, the lentivirus was produced with pCSII-EFp-CBR Luc-IRES2-Venus [13]. A Xenogen-IVIS 100-cooled CCD optical macroscopic imaging system was used for the bioluminescence imaging (BLI). All the images were analyzed with the Igor (WaveMetrics, Lake Oswego, OR) and Living Image software (Caliper Life Sciences), and the optical signal intensity was expressed as photon counts, in units of photons per second. The results were displayed as a pseudocolor photon count image superimposed on a gray-scale anatomic image. To quantify the measured luminescence, we defined a specific region of interest (ROI) that covered the area at and around the implanted cells. We used the same ROI for all the animals at all time points to ensure uniform data collection.

### Time-lapse imaging assay

Cells were transfected with fucci2;mAG-hGem(1/110), which labels nuclei in the S/G2/M phases [25]. The virus-infected (colored) cells were sorted by flow cytometry. Cells stably expressing the S/G_2_/M marker were grown in 96-well dishes in serum-free DMEM HAM/F12 (Sigma-Aldrich) supplemented with 1×B27 (Invitrogen), 20 ng/mL epidermal growth factor (EGF; Peprotech Invitrogen), and 20 ng/mL fibroblast growth factor-2 (FGF-2; Peprotech Invitrogen), at 37°C with 5% CO_2_. The cells were imaged for 96 hours in an incubator (Tokai Hit) on the microscope stage. Fifty-one images in the z-axis, both bright-field and single-color (green), were captured at 20-min intervals. An inverted microscope (IX-71, Olympus) was fitted with a Nipkow disc scanning confocal unit (CSU10, Yokogawa Electric Corp.), EM-CCD camera (iXON BV-887, Andor), filter wheel, and z motor (Mac5000, Ludl Electronic Products). As our imaging device has an attached auto xy stage (Sigma Koki), several spheres can be monitored in one assay. Device control and image analysis were performed using MetaMorph software (Universal Imaging).

Self renewing sphere formation was observed by time-lapse imaging microscopy.

## Supporting Information

Figure S1 **The spheres were dissociated, and the dissociated cells were fixed to coverslips and stained for PH3 in GM1605 cells.**
(TIF)

Figure S2 **Colonies were dissociated, and the cells were fixed to coverslips and stained for for cleaved Caspase-3 in GM1605 cells.**
(TIF)

Figure S3 **Low passage cells from glioblastoma patients (cell density of 2×10^3^ cells/well) were assessed for cell proliferation by cell counting.** The total cell count in the *MSI1*-KD-treated groups on day 15 was reduced by 39% in the GM1605 cells, as compared to the control groups.
(TIF)
