# Correction: RNA-Binding Protein Musashi1 Modulates Glioma Cell Growth through the Post-Transcriptional Regulation of Notch and PI_3_ Kinase/Akt Signaling Pathways

**DOI:** 10.1371/annotation/ab178792-f423-4aaf-8c26-950a54b8fcca

**Published:** 2012-07-10

**Authors:** Jun Muto, Takao Imai, Daisuke Ogawa, Yoshinori Nishimoto, Yohei Okada, Yo Mabuchi, Takeshi Kawase, Akio Iwanami, Paul S. Mischel, Hideyuki Saya, Kazunari Yoshida, Yumi Matsuzaki, Hideyuki Okano

There was an error in the author contributions designations. JM, TI, and HO conceived and designed the experiments. 

