# Correction: RNA-Binding Protein Musashi1 Modulates Glioma Cell Growth through the Post-Transcriptional Regulation of Notch and PI_3_ Kinase/Akt Signaling Pathways

**DOI:** 10.1371/annotation/4c24fcf6-cf55-42f7-aa0d-3984048a2d16

**Published:** 2013-04-25

**Authors:** Jun Muto, Takao Imai, Daisuke Ogawa, Yoshinori Nishimoto, Yohei Okada, Yo Mabuchi, Takeshi Kawase, Akio Iwanami, Paul S. Mischel, Hideyuki Saya, Kazunari Yoshida, Yumi Matsuzaki, Hideyuki Okano

There were numerous errors in the article.

In the lower panel of figure 1A (bar graphs), the values for KNS42, U251MG, Daoy, GM1600, GM1605, GM97 and hNSC should be 35, 104, 104, 72, 125, 144 and 100 rather than 40, 115, 156, 194, 218, 102 and 100, respectively.

In Figure5H, the indication of y-axis should be ‘Rate of PI positive cells’ rather than ‘Rate of PI negative cells’.

In the text, there were errors regarding figure number as follows (Page numbers based on PDF):

Page3 left column paragraph “MSI1-KD inhibits the growth of glioblastoma xenografts”

line11; 'Fig. 2' should be 'Fig. 3',

line14; 'Fig. 2A and B' should be 'Fig. 3A and B',

line 16; 'Fig. 2C' should be 'Fig. 3C',

line20; 'Fig. 2D' should be 'Fig. 3D',

line 22; Both '2F-a' should be '3F-a',

line 22; Both '2F-b' should be '3F-b',

line25; 'Fig. 2G-b, d' should be 'Fig. 3G-b, d’,

line27; 'Fig. 3G-a, c' should be 'Fig. 2G-a, c'.

Page3 right column paragraph ‘Treatment with MSI1-KD induces mitosis and G2/M arrest in spheres of glioblastoma cells’ line3, 'Fig. 2' should be 'Fig. 3', and 'Fig. 3' should be 'Fig. 2'.

Page12 left column paragraph “Virus production” line 7, '80ml' should be 80 (micro) l'. 

