# Supplementary figures and images for: RNA-Binding Protein Musashi1 Modulates Glioma Cell Growth through the Post-Transcriptional Regulation of Notch and PI3 Kinase/Akt Signaling Pathways
Source: PLoS One. 2012 Mar 12;7(3):e33431. doi: 10.1371/journal.pone.0033431 (PMC3299785; doi:10.1371/journal.pone.0033431)

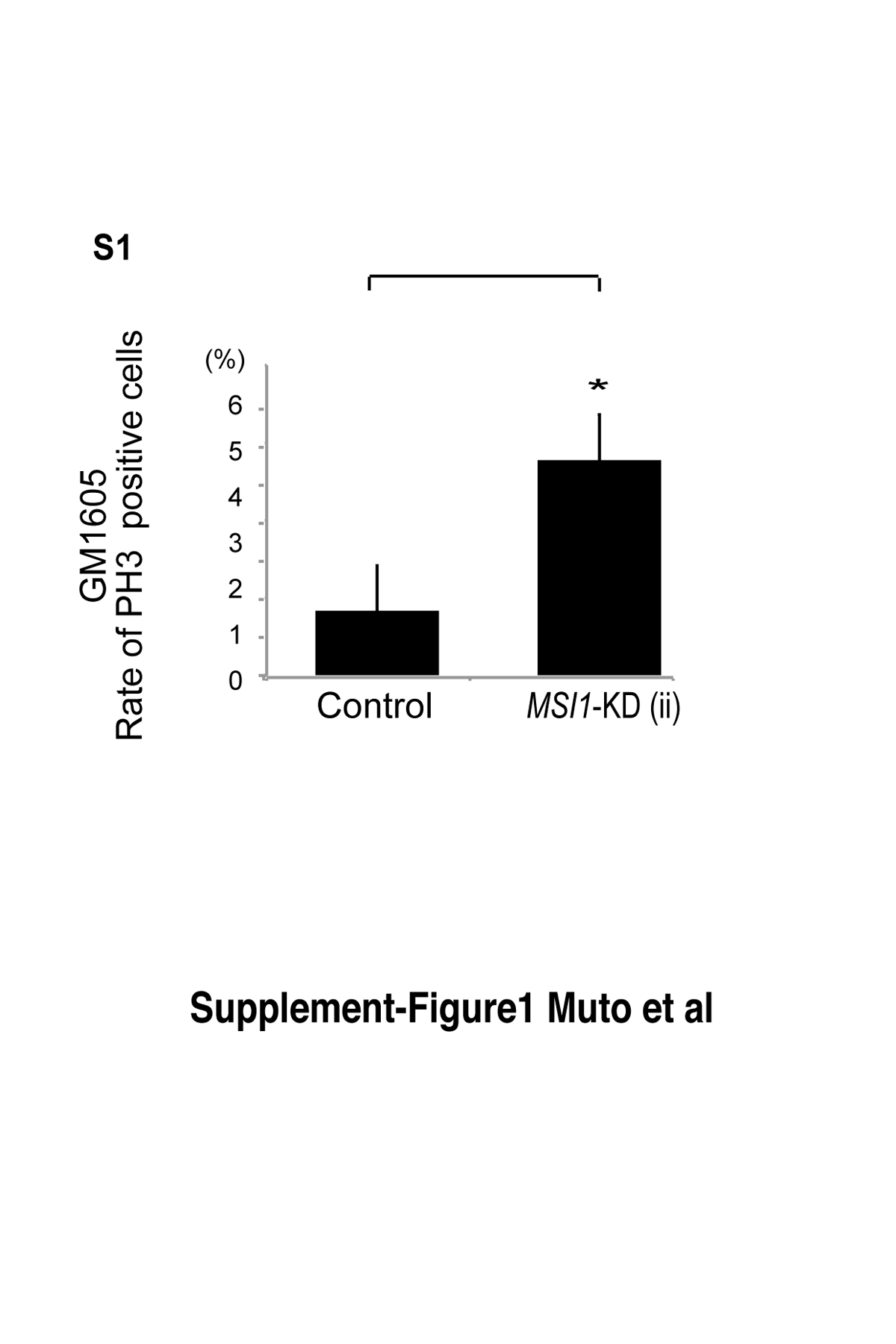

Supplement: Figure S1 — The spheres were dissociated, and the dissociated cells were fixed to coverslips and stained for PH3 in GM1605 cells. (TIF) [file pone.0033431.s001.tif]

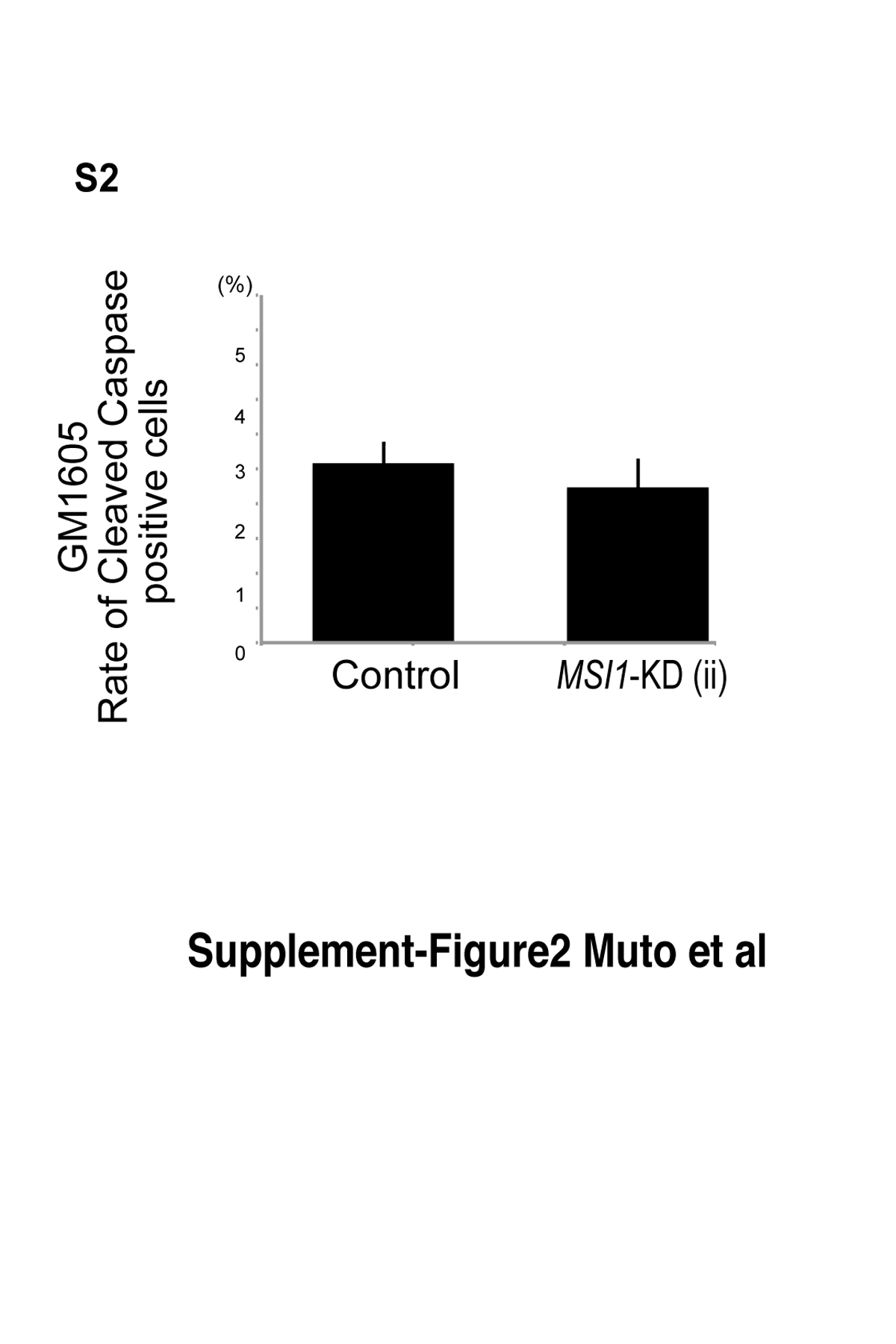

Supplement: Figure S2 — Colonies were dissociated, and the cells were fixed to coverslips and stained for for cleaved Caspase-3 in GM1605 cells. (TIF) [file pone.0033431.s002.tif]

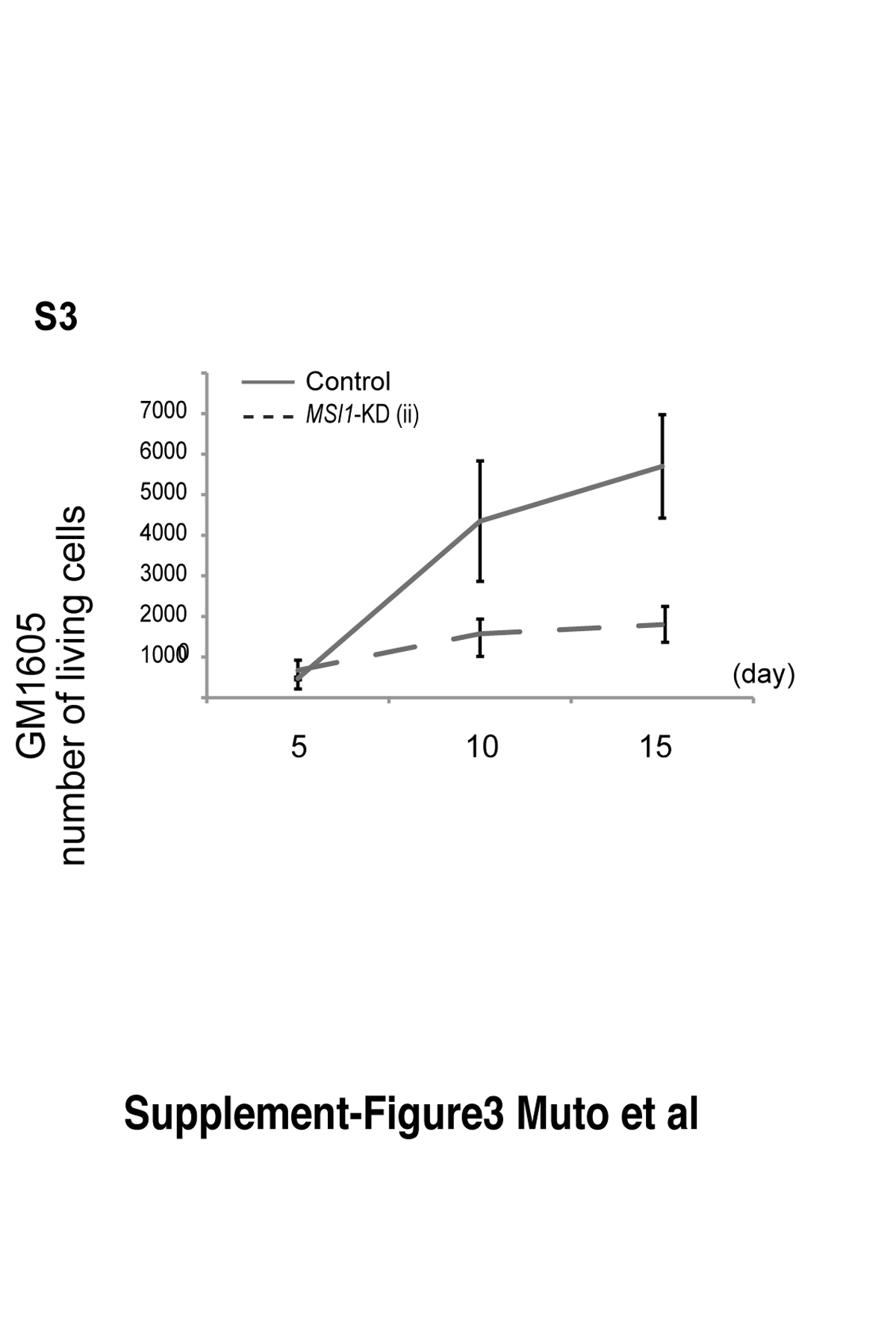

Supplement: Figure S3 — Low passage cells from glioblastoma patients (cell density of 2×10^3^ cells/well) were assessed for cell proliferation by cell counting. The total cell count in the MSI1-KD-treated groups on day 15 was reduced by 39% in the GM1605 cells, as compared to the control groups. (TIF) [file pone.0033431.s003.tif]
